# Drug-Induced Chylothorax During Chemotherapy With Ramucirumab and Paclitaxel for Advanced Gastric Cancer

**DOI:** 10.7759/cureus.77867

**Published:** 2025-01-23

**Authors:** Kenichi Sawa, Kohei Hayashi, Yuki Sonoda, Tomonori Araki, Takuya Honda

**Affiliations:** 1 Gastroenterology and Hepatology, Nagasaki University Graduate School of Biomedical Sciences, Nagasaki, JPN

**Keywords:** chylothorax, gastric cancer, lymphoscintigraphy, paclitaxel, ramucirumab

## Abstract

We encountered a rare case of ramucirumab (RAM)-induced chylothorax that resolved after treatment with a thoracic drain in a 75-year-old woman diagnosed with a HER2-positive advanced gastroduodenal carcinoma. Two weeks after initiating RAM and paclitaxel (PTX) treatment, a pleural fluid examination revealed chylothorax. Treatment with a thoracic drain was initially performed; however, continuous drainage was maintained even after the drain was placed. Since no leakage was observed on lymphoscintigraphy, she was treated without surgery or interventional radiology. The drainage volume gradually decreased; the patient was discharged after removing the thoracic drain. PTX alone was readministered, and chylothorax recurrence was not observed. We emphasized the possibility that RAM can cause chylothorax and that lymphoscintigraphy is useful for selecting treatment.

## Introduction

Chylothorax involves the leakage of lymphatic fluid into the thoracic cavity due to damage or obstruction of the chest ducts. Trauma is the most common cause of chylothorax [1]. In contrast, non-traumatic chylothorax is often caused by lymphatic invasion due to malignancy, cirrhosis, or infection [1,2]. Drug-induced chylothorax is rare, with only one reported case possibly involving ramucirumab (RAM) [3]. Other drugs, such as the tyrosine kinase inhibitor dasatinib, have been reported to cause this condition, although the detailed mechanism and incidence are unknown [4]. A criterion for selecting conservative or invasive treatment for non-traumatic chylothorax has not been established. Gastric cancer is common among East Asians, both men and women, and as cancer progresses, it is often accompanied by pleural effusion and ascites [5]. Based on the results of the RAINBOW study, RAM plus paclitaxel (PTX) therapy plays an important role in second-line chemotherapy for distant metastasis or recurrent gastric cancer [6]. Although RAM is an angiogenesis inhibitor, one of its action points, vascular endothelial growth factor receptor 2 (VEGFR-2), is also thought to inhibit lymphangiogenesis [7,8]. In this report, we describe a case of drug-induced nasopharyngeal effusion in a non-traumatic chylothorax that was thought to be caused by RAM. The absence of leakage on lymphoscintigraphy helped guide the decision not to perform surgery or radiological interventions. This study emphasized the usefulness of lymphoscintigraphy in determining treatment strategies and the importance of differentiating chylothorax from pleural effusion occurring during RAM administration.

## Case presentation

**Figure 1:**
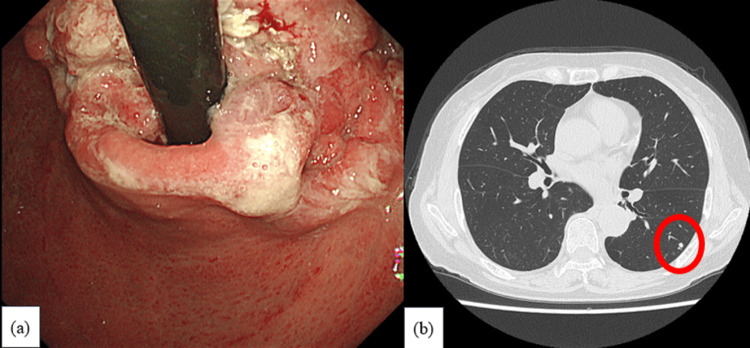
Imaging findings at inital visit (a) Esophagogastroduodenoscopy revealing gastric cardia cancer; (b) Computed tomography revealing a small metastasis in the left lower lobe (highlighted by a circle).

A 75-year-old woman was diagnosed with advanced HER2 3+ gastroduodenal adenocarcinoma, staged as cT4aN3M1 (lymphatic and pulmonary metastases) (Figure 1 and Table 1).

**Table 1:** Laboratory data at initial visit Blood tests at the time of the initial visit showed a mild increase in inflammatory response and slight anemia, although no increase in tumor markers. WBC: white blood cells; Hb: hemoglobin; PLT: platelet; AST: aspartate aminotransferase; ALT: alanine aminotransferase; Cr: creatinine; CRP: C-reactive protein; CA 19-9: cancer antigen 19-9

Laboratory data	Value	Reference range
WBC (/μL)	3500	3300-8600
Hb (g/dL)	11.0	11.6-14.8
PLT (/μL)	209000	158000-348000
Total protein (g/dL)	6.2	6.6-8.1
Albumin (g/dL)	3.1	4.1-5.1
AST (IU/L)	55	13-30
ALT (IU/L)	49	7-23
Lactate dehydrogenase (IU/L)	284	124-222
Cr (mg/dL)	0.52	0.46-0.79
CRP (mg/dL)	0.21	0.00-0.14
Total cholesterol (mg/dL)	173	142-248
Triglyceride (mg/dL)	80	30-117
Carcinoembryonic antigen (U/L)	2.6	<5.0
CA 19-9 (U/L)	<2.0	<37.0

**Figure 2:**
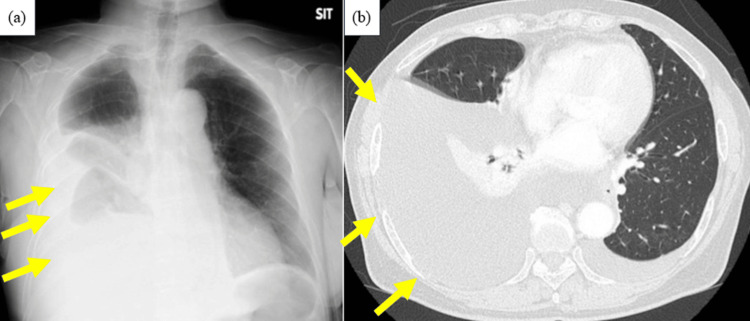
Radiography and computed tomography at emergency hospitalization (a) Radiograph and (b) computed tomography showing a right-sided predominant pleural effusion (indicated by arrows).

She had no previous history of surgery, trauma, recent esophagogastroduodenoscopy, or endoscopic ultrasonography procedures with fine-needle biopsy or other interventions. She had no family history of cancer and no radiation exposure. Despite six courses of capecitabine, oxaliplatin, and trastuzumab, disease progression was observed. Hence, she was started on RAM and PTX therapy. Two weeks later, she developed dyspnea and required hospital admission. This was five months after the initial diagnosis. Plain chest radiography and computed tomography (CT) revealed a right-sided predominant pleural effusion (Figure 2), for which diagnostic thoracentesis was performed.

**Figure 3:**
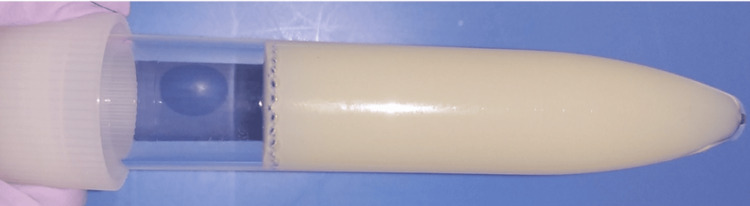
Appearance of the pleural fluid The pleural fluid appeared milky-white in color.

The pleural fluid was milky-white in color with a triglyceride count of 465 mg/dL (Figure 3 and Table 2).

**Table 2:** Laboratory test of the pleural fluid The triglyceride count of the pleural fluid is 465 mg/dL. There are no findings to support malignancy. CA 19-9: cancer antigen 19-9

Parameter	Value	Reference range
Triglyceride (mg/dL)	465	<110
Total cholesterol (mg/dL)	47	45-60
Total protein (g/dL)	1.5	1.0-2.0
Albumin (g/dL)	1.1	> serum albumin - 1.2
Lactate dehydrogenase (IU/L)	66	≤60% serum concentration
Adenosine deaminase (U/L)	4.2	<40
Carcinoembryonic antigen (U/L)	0.7	<5.0
CA 19-9 (U/L)	<2.0	<37.0
Culture	Negative	-
Cytology	Class Ⅱ	-

No signs of malignancy were observed on pleural fluid cytology or histology. No growth was seen on the pleural fluid culture, and no signs of infection were observed. Contrast-enhanced CT of the chest revealed no primary lung cancer or metastatic lung enlargement. Moreover, no malignant tumors, central venous obstruction, or thrombosis were observed.

**Figure 4:**
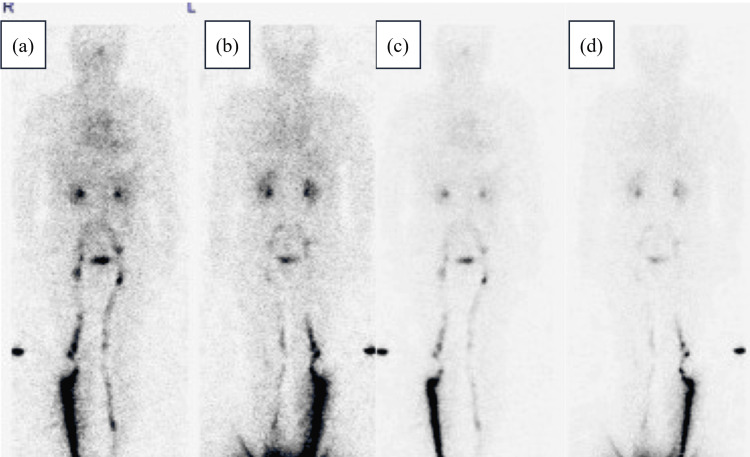
Lymphoscintigraphy on the 10th hospital day Lymphoscintigraphy performed immediately (a) anterior view, (b) posterior view, and 30 minutes later (c) anterior view, (d) posterior view. The imaging shows no obvious lymphatic leaks.

Due to persistent dyspnea, a thoracic drain was placed on the third day of admission for symptomatic improvement. Although pleural fluid continued to drain, lymphatic scintigraphy performed on the 10th hospital day showed no obvious leaks (Figure 4).

**Figure 5:**
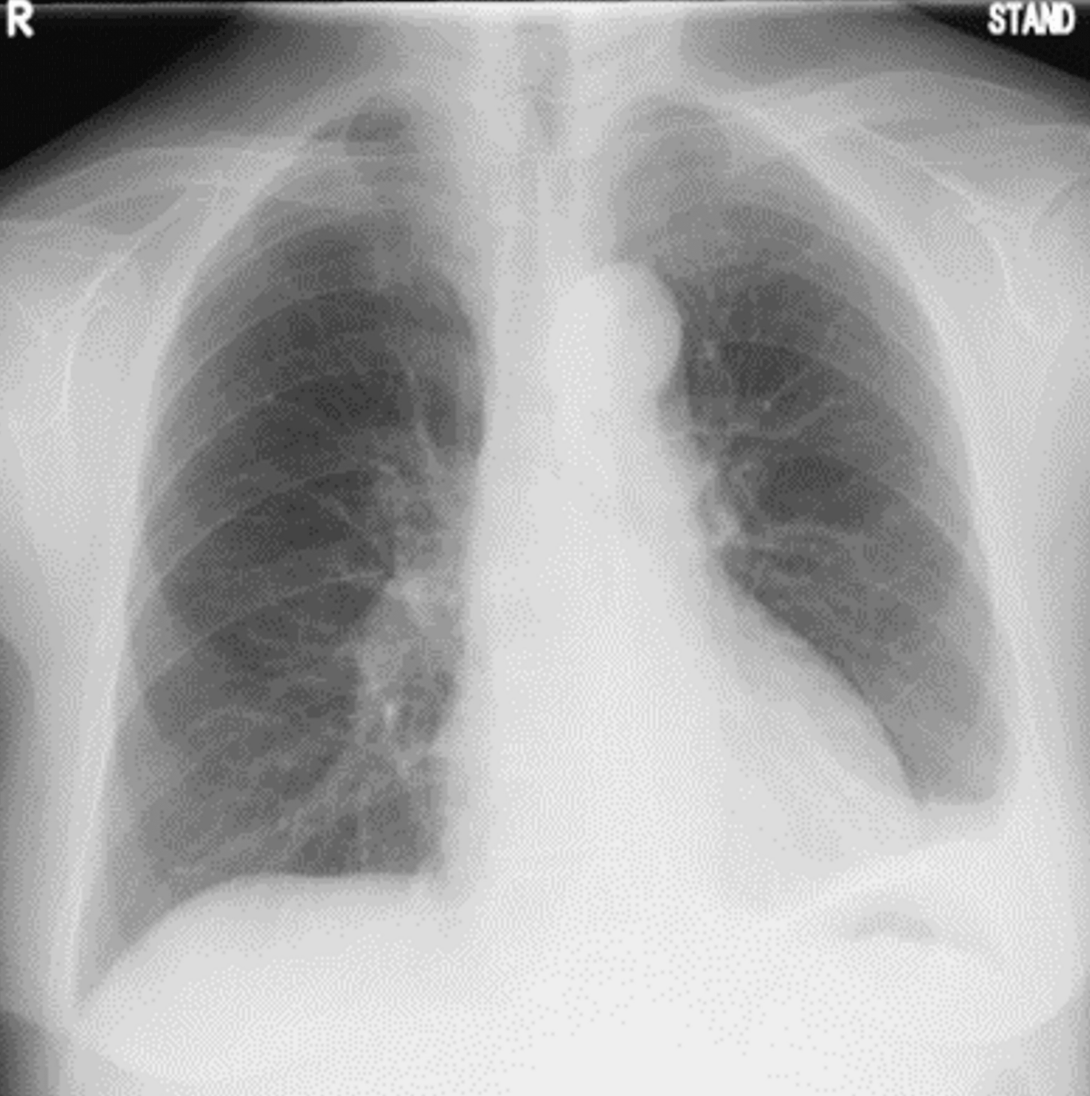
Radiography on the 13th hospital day The pleural effusion in the right lung has disappeared.

The patient was managed without surgery or radiological interventions. The drainage volume gradually decreased, and she did not develop symptoms of respiratory distress. The thoracic drain was successfully removed on the 13th hospital day (Figure 5).

**Figure 6:**
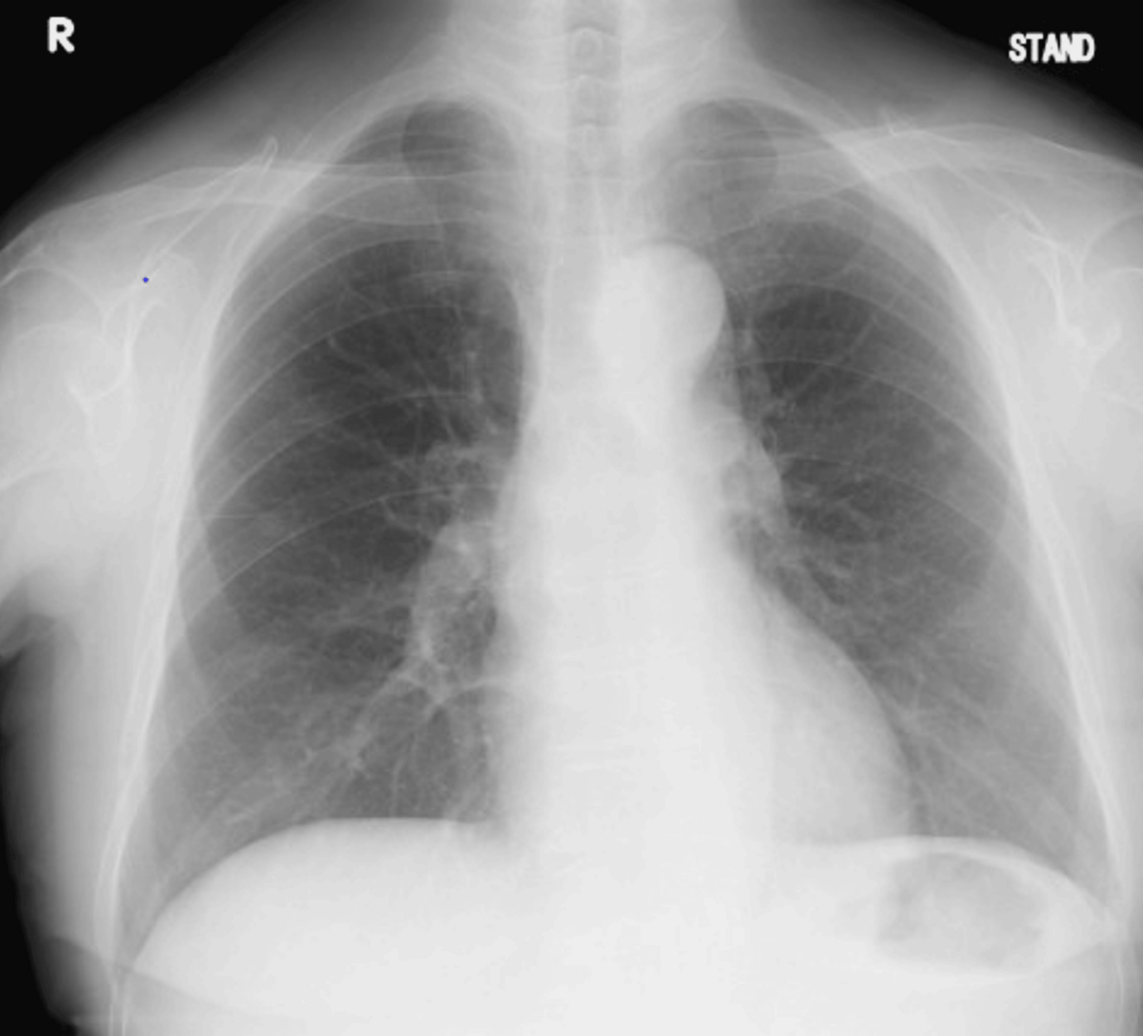
Radiography two months after onset of chylothorax There is no relapse of chylothorax.

Her pleural effusion improved, and she was discharged home on her 24th hospital day. PTX alone was readministered, and no recurrence of chylothorax was observed. Although the tumor subsequently increased, there was no recurrence of chylothorax (Figure 6).

A year and a half later, she died from the progression of cancer. All procedures followed were performed in accordance with the ethical standards laid down in the 1964 Declaration of Helsinki and its later amendments.

## Discussion

This case report highlights two important clinical implications. First, RAM may cause non-traumatic chylothorax, and second, lymphoscintigraphy is considered to be useful for determining the course of treatment for drug-induced chylothorax. Only one case of chylothorax during the combination of RAM and docetaxel (DTX) for small-cell lung cancer has been reported [3]. The incidence of chylothorax during RAM administration is unknown, as it has not been clearly demonstrated in clinical trials [6,9-11]. The lack of reports of real-world data may be due to publication bias, and it cannot be denied that disease progression occurring at the same time as the appearance of chylothorax may have been overlooked. However, in most cases, a drug-induced condition can only be diagnosed by exclusion unless the patient is re-administered, and it is difficult to prove a causal relationship based on previous reports, especially for rare side effects. RAM is a VEGFR-2 inhibitor, and VEGFR-2 is a receptor that induces angiogenesis, but also acts on lymphangiogenesis, so it was clinically determined that it was the cause of chylothorax [7,8]. Since PTX administration after chylothorax treatment was not problematic, a drug-induced PTX-induced chylothorax was ruled out. In addition, although the tumor subsequently increased, there was no recurrence of chylothorax as shown in Figure 6, and a neoplastic cause was also ruled out. We, therefore, consider that this study has educational value in showing that the effects of RAM should be considered in the differential diagnosis when chylothorax occurs during RAM administration.

First, RAM may cause non-traumatic chylothorax. RAM is a standard chemotherapy drug for gastric cancer [6], colorectal cancer [9], hepatocellular carcinoma [10], and non-small-cell lung cancer [11]. RAM is a fully human monoclonal IgG antibody that specifically binds to VEGFR-2 [12]. RAM inhibits the binding of VEGF-A, VEGF-C, and VEGF-D to VEGFR-2 [13]. VEGFR-3 is expressed on lymphatic endothelial cells (LECs), but VEGFR-2 also plays an essential role in lymphangiogenesis regulation, and cooperative signaling of VEGFR-2 and VEGFR-3 has been reported to be required for lymphatic migration and proliferation [6,7]. Thus, lymphangiogenesis may be induced by the VEGF-C and VEGF-D/VEGFR-2 and VEGFR-3 pathways. In the present case, RAM-induced inhibition of lymphangiogenesis through VEGFR-2 blockade, followed by damage to LECs, may have contributed to the development of chylothorax.

Second, the study highlights lymphoscintigraphy as a non-invasive tool to assess lymphatic leaks, aiding in the decision to pursue conservative treatment without surgical intervention. As shown in Figure 4, no leaks were observed. Treatment options for chylothorax include surgical treatment and radiologic interventions. The treatment without surgical treatment or radiologic interventions for traumatic chylothorax includes continuous thoracic drainage, diet (fat-restricted diet and central venous nutrition with fasting), subcutaneous octreotide injections, and pleurodesis [1]. Its invasive treatment includes thoracic duct ligation, suture of the leak, thoracic duct venous shunting, and embolization with lymphangiography [1]. However, the exact criteria for selecting invasive treatment in cases of non-traumatic chylothorax remain unestablished. In the present case, treatment with thoracic drainage was selected as the initial treatment. When considering whether to continue treatment or add invasive treatment, lymphoscintigraphy may be used to screen for leakage points. The patient continued to drain fluid from the thoracic drain, but the fluid drained gradually decreased as shown in Figure 5, and no point of leakage was observed as shown in Figure 4. Therefore, she underwent treatment without surgical treatment or radiologic interventions. Lymphoscintigraphy has been reported to be useful for identifying leakage points in traumatic chylothorax [14]. In drug-induced chylothorax, when considering whether to discontinue the causative drug or add surgical treatment or radiologic interventions, if no obvious leak points are identified on lymphoscintigraphy, treatment without surgical treatment or radiologic interventions may be done.

In this case, the patient developed non-traumatic chylothorax while receiving RAM and PTX for advanced gastric cancer and started treatment without surgical treatment or radiologic interventions. After hospital discharge, only PTX was resumed. Since the erosive pleural effusion did not recur, it was likely that it was drug-induced by RAM. A case has also been reported in which a patient with non-small cell lung cancer had an accumulation of chylous ascites and chylothorax during RAM and DTX therapy. However, the chylous ascites and chylothorax improved when RAM and DTX were simultaneously discontinued [3]. In that case, RAM and DTX were suspected drugs. DTX and PTX are both taxane anticancer drugs, although this case did not relapse even after resuming PTX, and RAM can also inhibit lymphangiogenesis through inhibition of VEGFR-2, so we judged that the disease was caused by RAM. Other reports of drug-induced erosive pleural effusions include a case report of drug-induced chylothorax caused by dasatinib in a patient with chronic myelogenous leukemia; however, the detailed mechanism of action is unknown [4]. Treatment without surgical treatment or radiologic interventions for drug-induced chylothorax includes discontinuing the causative agent and administering furosemide, steroids, and herbal medicines as drug therapy [15]. In most cases, effusion drainage is necessary when the patient is symptomatic, as in the present case. As shown in Figure 2, radiography and CT scans revealed pleural effusion in the right lung and she developed dyspnea, so a drain was inserted in this case. However, prolonged drainage should be avoided as it can lead to immunodeficiency, malnutrition, severe electrolyte disturbances, and increased morbidity and mortality [2]. In this case, when deciding when to remove the thoracic drain, we determined that drain removal and discontinuation of the causative agent alone may relieve the lactic pleural effusion, as there were no obvious leak points on lymphoscintigraphy.

There were some limitations. First, pathological confirmation of surgical specimens is necessary to completely rule out the influence of pulmonary metastasis or pleural dissemination, although this was lacking in this case because the patient improved. However, because the patient improved with thoracic drainage alone, there was no subsequent recurrence of chylothorax, and there was no increase in pulmonary metastasis at the time of onset, we considered that the influence of pulmonary metastasis or pleural dissemination is unlikely. Second, the pharmacological interaction between RAM and PTX may have caused the chylothorax; however, the mechanism underlying PTX-induced chylothorax is unknown. Further case studies are required to confirm them and analyze the effects of different treatment options. Another option would be to study the effects of RAM on the lymphatic system in animal experiments.

## Conclusions

This case suggests that RAM induces chylothorax during drug administration. In cases of drug-induced chylothorax, treatment with a thoracic drain is initiated, and if there are no obvious leak points on lymphoscintigraphy, the chylothorax may improve without surgical treatment or radiologic interventions. When a patient with RAM develops chylothorax, the possibility of a drug-induced RAM-related condition should be considered in the differential diagnosis. However, in most cases, a drug-induced condition can only be diagnosed by exclusion unless the patient is re-administered, and it is difficult to prove a causal relationship based on previous reports, especially for rare side effects. Further studies are needed on incidence and drug interactions.
